# Plasticity of the anatomical traits of *Rhododendron* L. (Ericaceae) leaves and its implications in adaptation to the plateau environment

**DOI:** 10.1515/biol-2025-1116

**Published:** 2025-05-27

**Authors:** Wenwen Guo, Mecao Zhuo, Youzhi Bai, Jiangping Fang

**Affiliations:** School of Ecology and Environment, Tibet University, 850000, Xizang, China; Institute of Xizang Plateau Ecology, Xizang Agriculture & Animal Husbandry University, 860000, Xizang, China; Key Laboratory of Forest Ecology in Tibet Plateau, Ministry of Education, 860000, Xizang, China; Pai Town Agricultural and Animal Husbandry Comprehensive Service Center, Nyingchi, 86000, Xizang, China; Department of Resources and Environment, Xizang Agriculture & Animal Husbandry University, 860000, Xizang, China

**Keywords:** *Rhododendron* L., leaf anatomical traits, plasticity index, environmental adaptation

## Abstract

There is a variety of *Rhododendron* plants in the Tibetan plateau; yet, little is known about their variations in leaf anatomical traits and the implications for environmental adaptation. In this study, we investigated the anatomical traits of leaves in five *Rhododendron* L. species from Shergyla Mountain on the Tibetan plateau. The results showed that the five species have notable intraspecific and interspecific differences in the thickness of leaves, cuticle thickness, adaxial epidermis thickness, palisade parenchyma thickness, spongy parenchyma thickness, and tightness of leaf palisade parenchyma (*P <* 0.05). There are autocorrelations among these anatomical traits of the leaves, indicating the existence of synergistic changes. The interspecific variations in leaves’ anatomical structures illustrated their spectrum of plasticity to habitat heterogeneity. Our findings demonstrated that *Rhododendron* plants have developed typical ectopic leaves to adapt to harsh conditions of the Tibetan plateau.

## 1 Introduction

Leaves serve as the primary organs for transpiration, photosynthesis, and respiration in plant species. The morphology and anatomical traits of leaves link intimately to surrounding biotic and abiotic conditions and, consequently, exhibit different characteristics among species growing in different habitats, representing manifestations of species evolution and environmental adaptation [1,2]. Most studies addressed the anatomy of leaves in different plant species of different regions [3,4,5]; yet, less attention has been paid to the comparison of leaf anatomy traits of different species of the same genus and in the same region.

The leaves of plants adjust their morphology and anatomy in response not only to different regional growing conditions but also to changing environments of the same region [6]. In regions that are sensitive to climate change and ecological disturbances, plants exhibit a wide range of morphological and structural alterations in their leaves. The plasticity index of plant anatomical straits can be defined as a quantitative index that reflects the degree of change in plant anatomical traits in response to environmental variations. For instance, under high-light or arid conditions, plants may show an elevated plasticity index in leaf thickness. Thicker leaves can reduce the surface-to-volume ratio, minimizing water loss through transpiration [7]. Additionally, an increase in the density of palisade parenchyma cells, as indicated by a higher plasticity index, enhances light-harvesting efficiency [8]. The extent to which plants alter their leaf form and structure reflects leaf plasticity to environmental variation [9,10]. Such plasticity is an important ecological strategy for plants to cope with changing environments [11]. However, previous studies have rarely considered the relationship between leaf anatomy traits and plasticity in different plants of the same genus in the same region.

Leaf anatomical traits, including the tissue of epidermal, vein, and mesophyll, are sensitive and adaptable to environmental changes [12,13]. For instance, the epidermal parenchyma acts as the protective tissue of the leaf against adverse conditions, and epidermal parenchyma thickness could reflect the ability of the leaf for heat preservation and water adjustment [14]. The palisade parenchyma, which is the main site of photosynthesis, modulates leaf photosynthetic efficiency through changes in tissue porosity [15]. However, how the *Rhododendron* plant leaf anatomical traits function among themselves to adapt to the plateau environment is not yet known.

*Rhododendron* L. comprises the largest woody plant genus in the Northern Hemisphere, it belongs to Ericaceae, encompassing over 1,000 species [16]. China shelters approximately 600 *Rhododendron* species, and more than 20 species grow in Shergyla Mountain on the Tibetan plateau [17,18]. Previous studies of *Rhododendron* plants in Shergyla Mountain reported that the size of leaves, color of flowers, and size of seeds are the key indicators of these plants’ adaptability [19,20]. To date, it remains unclear to what extent different species of *Rhododendron* plants adjust their leaf morphology and anatomy traits to adapt to the changing conditions of the plateau.

Here, we tackle the above question from the perspective of leaf plasticity for ecological adaptation to cope with Tibetan plateau environments. Our study focuses on five species of *Rhododendron* plants with the objectives (1) are there synergistic features in the anatomical traits of *Rhododendron* leaves, (2) to explore leaf plasticity of *Rhododendron* and its linkage to adaptation to plateau environments.

## 2 Materials and methods

### 2.1 Study sites

The study was conducted in Shergyla Mountain, Nyingchi City, Tibet Autonomous Region (94°28′–94°51′E, 29°25′–29°57′N). The region experiences distinct wet and dry seasons, with average annual precipitation of 1,134 mm primarily falling from June to September (80%). Average annual relative humidity is 78.83%, evaporation is 544 mm, and temperature is −0.73°C. The warmest month (July) averages 9.23°C, and the coldest month (January) averages −13.98°C. Annual sunshine and frost-free periods are 1,151 h and 160 days, respectively [18,21]. Dominant arboreal species include *Abies georgei Orr* var. *smithii (Viguie et Gaussen) Cheng et* L. Shrub layer species comprise *Salix oritrepha* Schneid, *Ribes glaciale*, *Sorbus rehderiana* Koehne, *Potentilla fruticose* L., etc. Soils are predominantly mountain brown loam and brown acidic loam ranging from neutral to extremely acidic [18].

### 2.2 Materials

**Figure 1:**
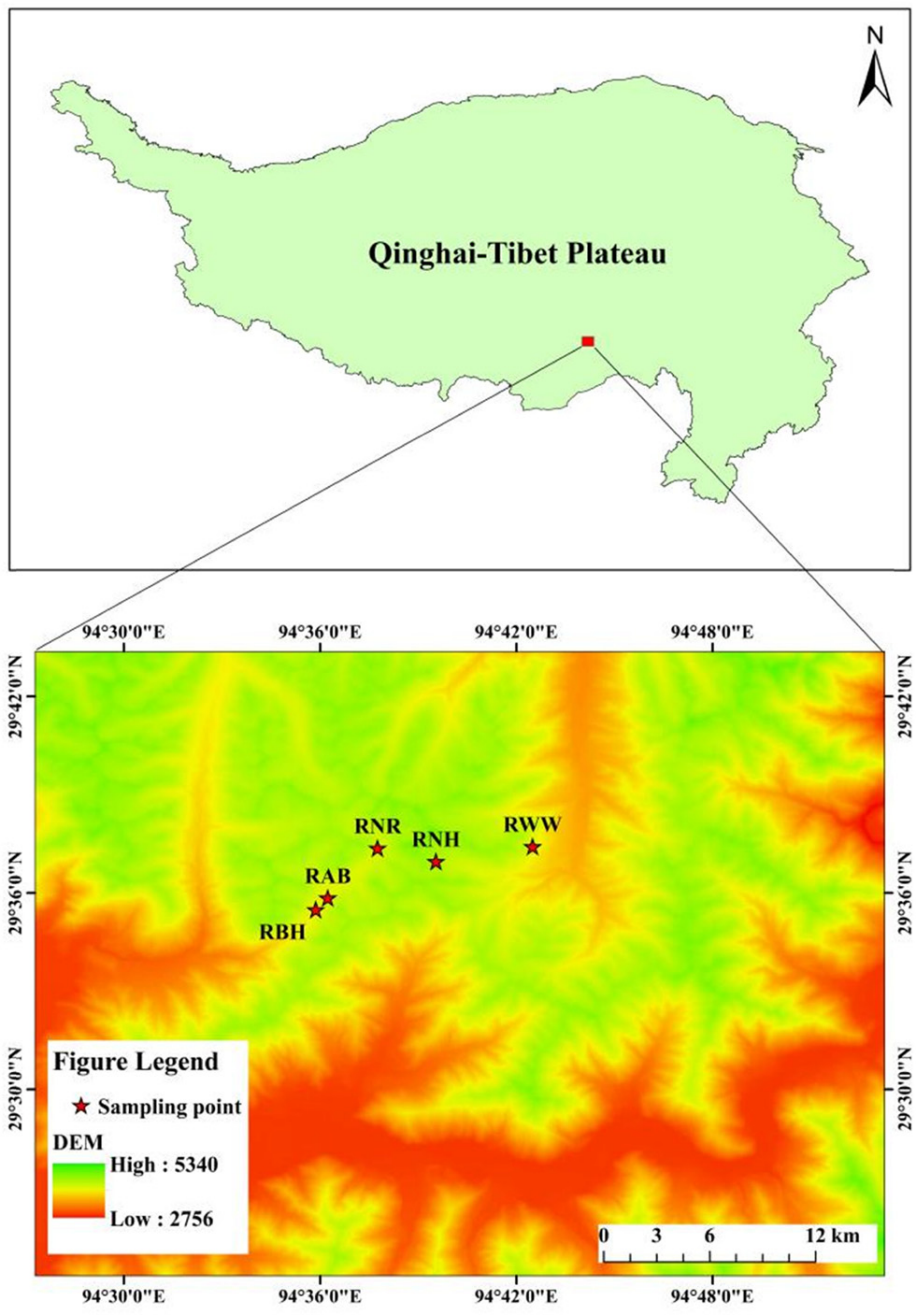
Information on sampling sites in the Shergyla Mountain, Note: RBH: *Rhododendron bulu*; RAB: *Rhododendron aganniphum*; RNR: *Rhododendron nyingchiense*; RNH: *Rhododendron nivale*; RWW: *Rhododendron wardii*.

In July 2023, a survey of *Rhododendron* was conducted in Shergyla Mountain, Tibet (Figure 1). Leaves were collected from five species: *Rhododendron bulu* Hutch., *Rhododendron wardii* W. W. Smith, *Rhododendron nyingchiense* R. C. Fang et S. H. Huang, *Rhododendron nivale* Hook. f., and *Rhododendron aganniphum* Balf. f. et K. Ward. For each species, a plot of 20 m × 20 m was established with simultaneous collection of geographic data including elevation, slope, latitude, longitude, and vegetation coverage (Table 1). At each sampling point, mature and healthy leaves were collected from three *Rhododendron* plants of similar developmental/growth status. Leaves were stored in fixation solution (5 ml formaldehyde, 5 ml acetic acid, 90 ml 50% ethanol, 5 ml glycerin) for later anatomical measurements [22].

**Table 1:** Basic information of the research sites

No.	Species	Longitudes	Latitude	Altitude (m)	Vegetation coverage (%)	Slope (°)
1	RBH	94°35′51.90″	29°35′29.31″	4,094.20	65	25
2	RAB	94°36′14.00″	29°35′50.92″	4,129.62	50	23
3	RNR	94°37′45.61″	29°37′22.16″	4,253.95	55	31
4	RNH	94°39′32.62″	29°36′57.96″	4,469.79	60	27
5	RWW	94°42′29.66″	29°37′25.15″	4,186.42	60	29

Note: RBH: *Rhododendron bulu*; RAB: *Rhododendron aganniphum*; RNR: *Rhododendron nyingchiense*; RNH: *Rhododendron nivale*; RWW: *Rhododendron wardii*.

### 2.3 Measurements of leaf anatomical traits

*Rhododendron* leaves were removed from the fixation solution and rinsed three times with distilled water, and 1 cm^2^ leaf fragments were cut with a razor blade. Samples were dehydrated through an ethanol series, cleared in xylene, embedded in paraffin, sectioned at 10 μm thickness, and stained with safranin O-fast green. Sections were sealed on slides with neutral gum [23]. Ten slides were prepared per species and sealed. A Nikon ECLIPSE 80i microscope (Nikon Corporation, Japan) and imaging system were used to observe and photograph slides under 2–3 random fields of view per slide.

Plant anatomical traits include the epidermis parenchyma thickness, palisade parenchyma thickness (μm) and spongy parenchyma thickness (μm), as well as certain markers of environmental adaptation, like the degree of midrib protuberant, the palisade parenchyma/spongy parenchyma ratio, ratio of palisade parenchyma to leaf thickness (%) and spongy parenchyma to leaf thickness ratio (%).

NIS-Elements software (Nikon Corporation, Japan) was used to measure the leaf anatomical traits, including cuticle thickness (μm), adaxial epidermal thickness (μm), palisade parenchyma thickness, spongy parenchyma thickness, abaxial epidermal thickness (μm), midrib vessel diameter, and leaf thickness (μm). Among them, 24 sets of data were measured for all indicators except for the midrib vessel diameter, which was measured in 15 sets of data.

### 2.4 Data analysis

To identify the relationships among the anatomical features of plant leaves, the subsequent metrics are computed following equations (1)–(4): ratio of palisade parenchyma to leaf thickness, spongy parenchyma-to-leaf thickness ratio, the palisade parenchyma/spongy parenchyma ratio, and degree of midrib protuberant. For each of the above metric, the plasticity index (PI) was calculated following equations (5) [24]. These measurements characterize inter- and intra-specific variation in phenotypic plasticity of leaf structure traits.

(1) \[\text{Ratio of palisade parenchyma to leaf thickness}=\text{palisade parenchyma thickness}/\text{leaf thickness}\times 100 \% ,]\]

(2) \[\text{Spongy parenchyma to leaf thickness ratio}=\text{spongy parenchyma thickness}/\text{leaf thickness}\times 100 \% ,]\]

(3) \[\text{Palisade parenchyma}/\text{spongy parenchyma ratio}\hspace{.25em}=\text{palisade parenchyma thickness}/\text{spongy parenchyma thickness,}]\]

(4) \[\text{Degree of midrib protuberant}=\text{midrib vessel diameter}/\text{leaf thickness,}]\]

(5) \[\text{PI}=\hspace{.25em}(\text{Maximum mean}-\text{minimum mean})/(\text{maximum mean}).]\]

We employed the Shapiro–Wilk test to check for normality. We choose one-way analysis of variance to evaluate differences in trait means among multiple species. Variability in leaf anatomical traits and correlation analysis was examined using IBM SPSS Statistics 26.0 (IBM, USA). Differences in metrics were compared using boxplots and radar chart created in Origin 2021 (OriginLab, USA). Correlation analysis was performed using the Pearson method.

## 3 Results

### 3.1 *Rhododendron* leaf anatomical structures

**Figure 2:**
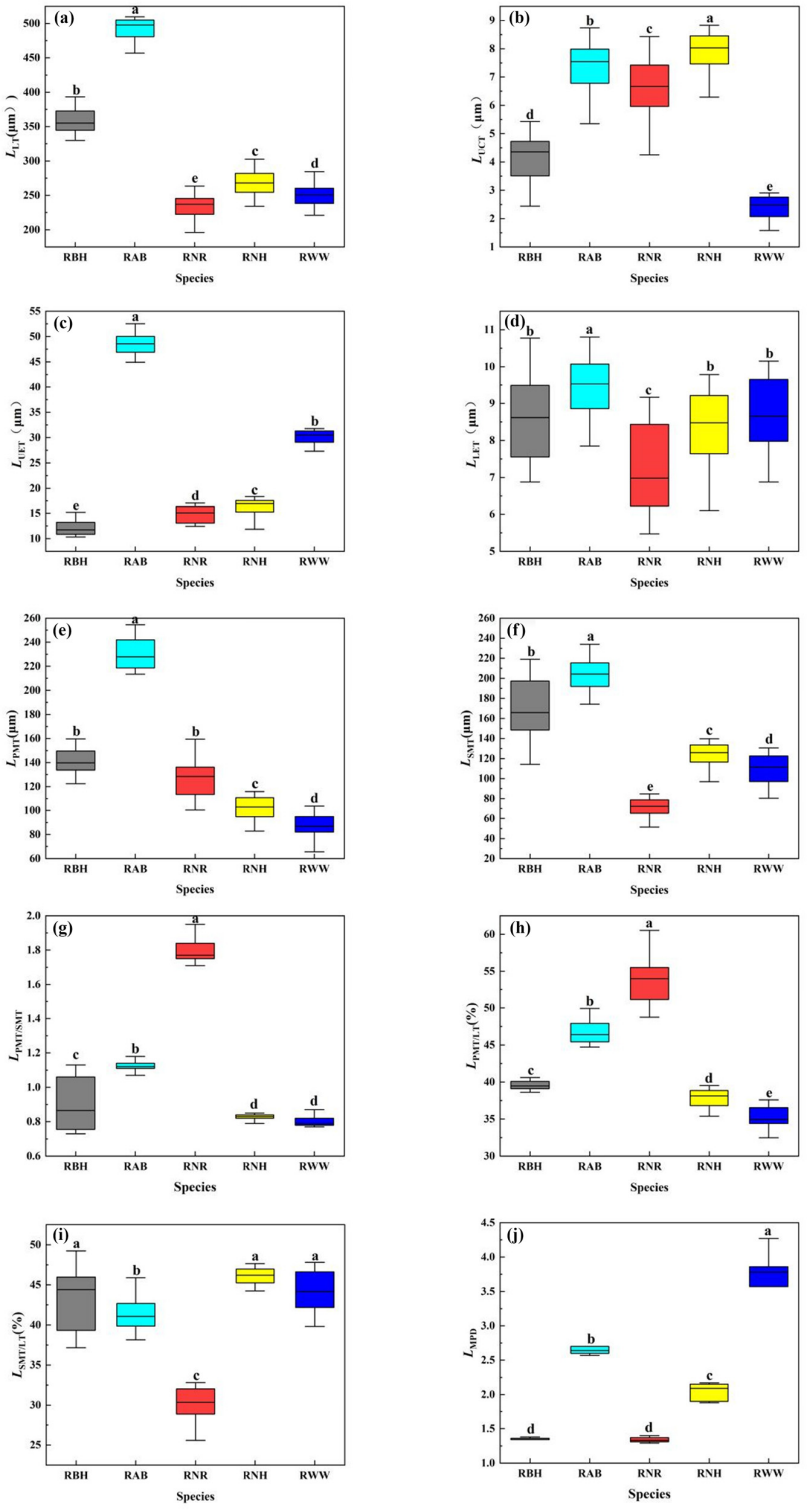
*Rhododendron* leaf anatomical structural values. Notes: Significant differences at the *P <* 0.05 level are indicated by lowercase letters in the same structure. *L*_LT_: leaf thickness; *L*_UCT_: cuticle thickness; *L*_UET_: adaxial epidermal thickness; *L*_LET_: abaxial epidermal thickness; *L*_PMT_: palisade parenchyma thickness; *L*_SMT_: spongy parenchyma thickness; *L*_PMT/SMT_: palisade parenchyma/spongy parenchyma ratio; *L*_PMT/LT_: ratio of palisade parenchyma to leaf thickness; *L*_*S*MT/LT_: spongy parenchyma to leaf thickness ratio; *L*_MPD_: degree of midrib protuberant. RBH: *Rhododendron bulu*; RAB: *Rhododendron aganniphum*; RNR: *Rhododendron nyingchiense*; RNH: *Rhododendron nivale*; RWW: *Rhododendron wardii*.

**Figure 3:**
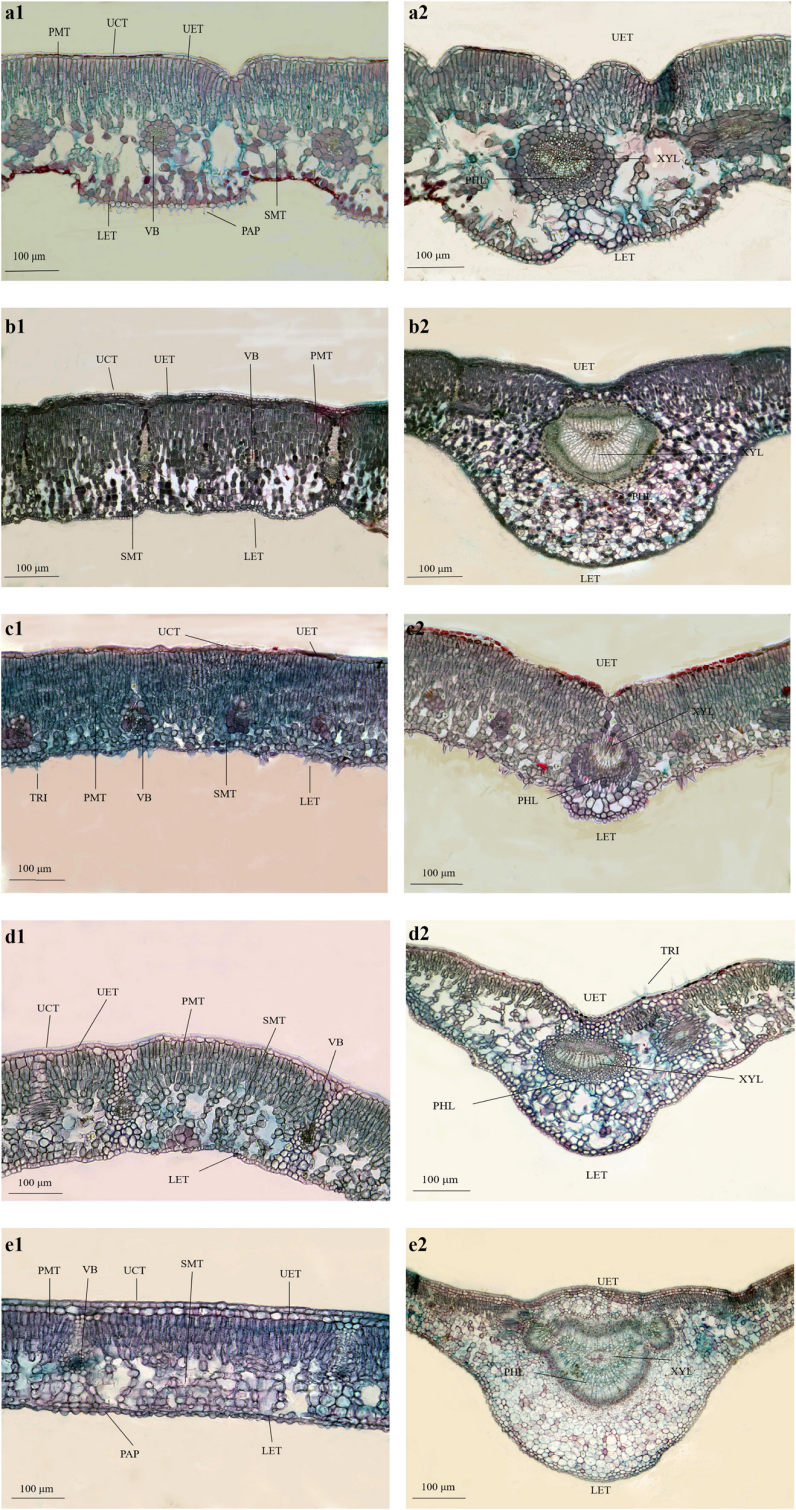
Leaves anatomical traits of five species of *Rhododendron*. Notes: (a1 and a2): *R. bulu*, (b1 and b2): *R. aganniphum*, (c1 and c2): *R. nyingchiense*, (d1 and d2): *R. nivale*, (e1 and e2): *R. wardii*. CUT: cuticle, UPE: adaxial epidermal, PP: palisade parenchyma, SP: spongy parenchyma, LOE: abaxial epidermal, PAP: papilla, XYL: xylem, PHL: phloem, VB: vascular bundle, TRI: Trichome.

Significant interspecific variation was observed for leaf thickness, cuticle thickness, adaxial epidermal thickness, palisade parenchyma thickness, spongy parenchyma thickness, and ratio of palisade parenchyma to leaf thickness (Figure 2, *P* < 0.05). Leaves of the five *Rhododendron* species from Shergyla Mountain exhibited typical bifacial morphology composed of veins, epidermis and mesophyll (Figure 3). Leaf thickness ranged from 235.51 to 492.06 μm among species (Figure 2a). *R. aganniphum* had the thickest leaves whereas *R. nyingchiense* had the thinnest leaves (Figure 2a).

Cuticle thickness varied between 2.38 and 7.92 μm across the five *Rhododendron* species (Figure 2b). All of the *Rhododendron* in this study has a subepidermal layer. The adaxial and abaxial epidermis of leaves consisted of closely arranged cells. *R. wardii* had two layers of cells in the adaxial epidermis and one layer in the abaxial epidermis (Figure 3e1). In contrast, *R. aganniphum* displayed one layer of cells in the abaxial epidermis and three layers in the adaxial epidermis (Figure 3b1). The remaining three species had a single cell layer in both the adaxial and abaxial epidermis (Figure 3a1, c1 and d1). Adaxial epidermal thickness ranged from 12.04 to 48.69 μm (Figure 2c) and abaxial epidermal thickness from 7.29 to 9.47 μm among species (Figure 2d). *R. aganniphum* exhibited the thickest adaxial and abaxial epidermis (Figure 2c and d). Distinct papillae structures were observed on epidermal cells of *R. bulu* and *R. wardii* (Figure 3a1 and e1), while trichomes were present on *R. nyingchiense* and *R. nivale* epidermis (Figure 3c1 and d2).

The leaf mesophyll is divided into palisade and spongy parenchyma. Palisade parenchyma, adjacent to the adaxial epidermis interior, comprised neatly aligned long columnar parenchyma cells. *R. bulu*, *R. nivale*, and *R. wardii* exhibited three palisade layers (Figure 3a1, d1, and e1), whereas *R. nyingchiense* had three to four layers (Figure 3c1) and RAB displayed four to five layers (Figure 3b1). *R. nyingchiense* showed the highest ratio of palisade parenchyma to leaf thickness (53.68%, Figure 2h) and palisade parenchyma/spongy parenchyma ratio (1.80, Figure 2g). Spongy parenchyma, adjacent to the inner side of abaxial epidermis, comprised irregular parenchyma cells with intercellular spaces. Its thickness ranged from 70.76 to 204.15 μm (Figure 2f). *R. nyingchiense* featured the thinnest spongy abaxial whereas *R. aganniphum* exhibited the thickest (Figure 2f).

Xylem and phloem elements within *Rhododendron* leaves demonstrated clear differentiation, with the primary vein well developed (Figure 3a2, b2, c2, d2, and e2). The degree of midrib protuberant varied from 1.35 to 3.64 among species (Figure 2j), as did the main vein diameter which ranged from 304.97 to 1324.97 μm. *R. aganniphum* exhibited the largest main vein diameter but a smaller degree of midrib protuberant (2.74) than *R. wardii*. In contrast, *R. nyingchiense* displayed the least developed vein diameter and smallest degree of midrib protuberant, though its main vein diameter was 4.34-fold thicker than *R. wardii*, but the degree of midrib protuberant of species *R. wardii* was 2.03-fold greater than that of *R. nyingchiense* (Figure 2j). Variation in midrib morphological plasticity potentially optimized hydraulic conductance in relation to habitat water availability.

### 3.2 Pearson correlation analysis of leaf anatomical structures

Pearson correlation analysis revealed some autocorrelation between leaf anatomical traits of *Rhododendron* species (Table 2). Spongy parenchyma to leaf thickness ratio exhibited a strong negative correlation with palisade parenchyma/spongy parenchyma ratio (*P* < 0.01) as well as a significant negative relationship with ratio of palisade parenchyma to leaf thickness (*P* < 0.05). Leaf thickness positively correlated with both palisade parenchyma thickness and spongy parenchyma thickness (*P* < 0.05). Spongy parenchyma thickness also correlated positively with abaxial epidermal thickness (*P* < 0.05). Palisade parenchyma/spongy parenchyma ratio positively correlated with ratio of palisade parenchyma to leaf thickness (P < 0.05). All other pairwise correlations between traits were insignificant (*P* > 0.05).

**Table 2:** Pearson correlation analysis of leaf anatomical traits in *Rhododendron*

	*L*_UCT_	*L*_UET_	*L*_LET_	*L*_PMT_	*L*_SMT_	*L*_PMT/SMT_	*L*_PMT/LT_	*L*_SMT/LT_	*L*_MPD_
*L*_LT_	0.279	0.692	0.789	0.936*	0.949*	−0.142	0.134	0.243	0.088
*L*_UCT_	1	0.093	−0.126	0.434	0.154	0.359	0.509	−0.252	−0.409
*L*_UET_		1	0.738	0.678	0.617	−0.120	0.064	0.121	0.690
*L*_LET_			1	0.557	0.902*	−0.657	−0.449	0.692	0.567
*L*_PMT_				1	0.780	0.208	0.469	−0.106	−0.047
*L*_SMT_					1	−0.439	−0.175	0.533	0.176
*L*_PMT/SMT_						1	0.958*	−0.988**	−0.493
*L*_PMT/LT_							1	−0.912*	−0.488
*L*_SMT/LT_								1	0.397

Notes: **indicates a significant correlation at the 0.01 level, and *indicates a significant correlation at the 0.05 level. *L*_LT_: leaf thickness; *L*_UCT_: cuticle thickness; *L*_UET_: adaxial epidermal thickness; *L*_LET_: abaxial epidermal thickness; *L*_PMT_: palisade parenchyma thickness; *L*_SMT_: spongy parenchyma thickness; *L*_PMT/SMT_: palisade parenchyma/spongy parenchyma ratio; *L*_PMT/LT_: ratio of palisade parenchyma to leaf thickness; *L*_*S*MT/LT_: spongy parenchyma to leaf thickness ratio; *L*_MPD_: degree of midrib protuberant.

### 3.3 Plasticity index of leaf anatomical traits in *Rhododendron*

**Figure 4:**
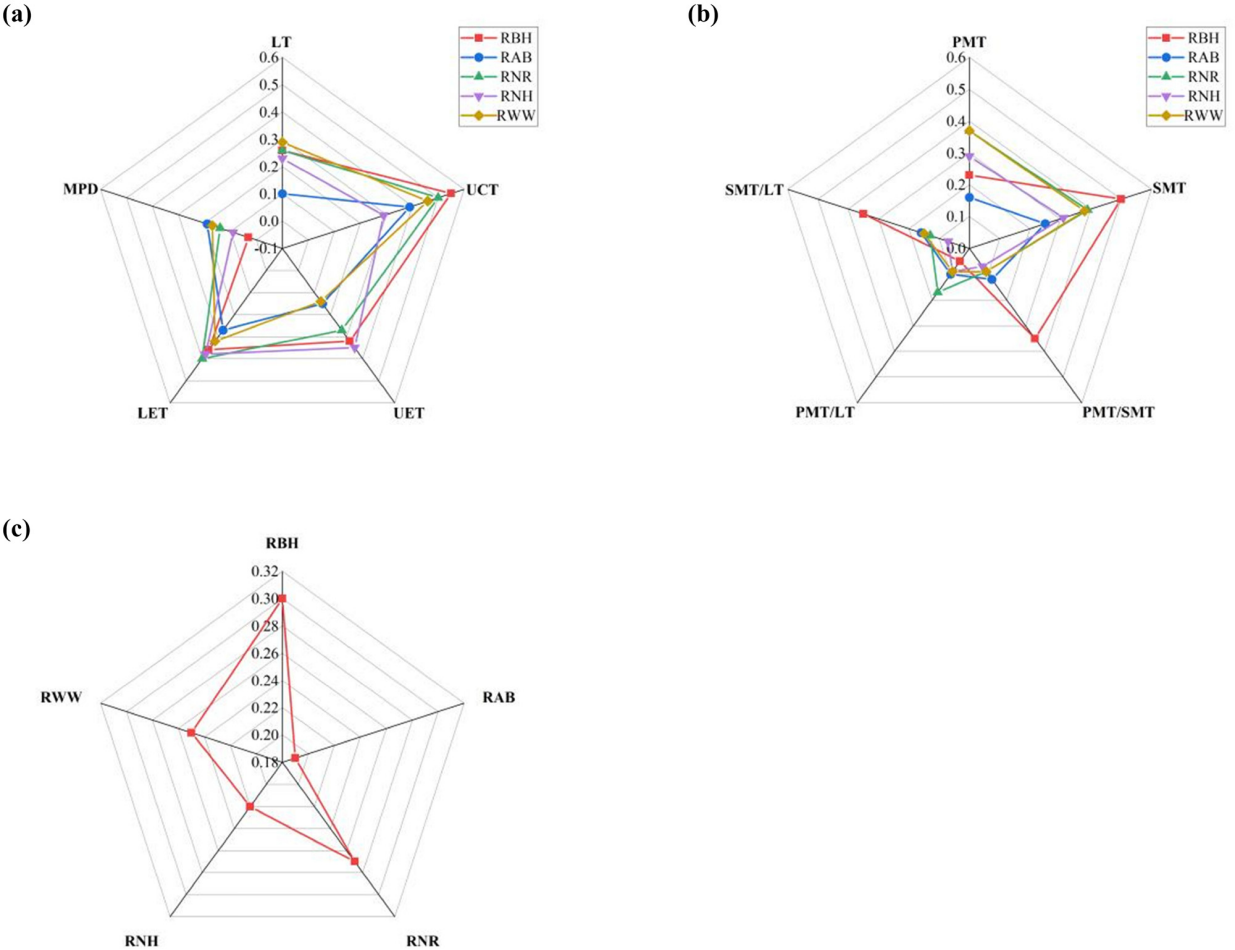
Plasticity index of leaf anatomical traits in *Rhododendron,* Note: LT: leaf thickness; UCT: cuticle thickness; UET: adaxial epidermal thickness; LET: abaxial epidermal thickness; PMT: palisade parenchyma thickness; SMT: spongy parenchyma thickness; PMT/SMT: palisade parenchyma/spongy parenchyma ratio; PMT/LT: ratio of palisade parenchyma to leaf thickness; SMT/LT: spongy parenchyma to leaf thickness ratio; MPD: degree of midrib protuberant. RBH: *Rhododendron bulu*; RAB: *Rhododendron aganniphum*; RNR: *Rhododendron nyingchiense*; RNH: *Rhododendron nivale*; RWW: *Rhododendron wardii*.

The leaf anatomical traits had a plasticity index ranging from 0.10 to 0.44 (Figure 4). The plasticity indices for cuticle thickness, spongy parenchyma thickness, and abaxial epidermal thickness were greater, while those for the ratio of palisade parenchyma to leaf thickness, degree of midrib protuberant, and palisade parenchyma/spongy parenchyma ratio were lower (Figure 4a and b). Overall, cuticle thickness, spongy parenchyma thickness, and abaxial epidermal thickness had greater plasticity index (Figure 4a and b). For the five species of *Rhododendron*, the order of magnitude of the plasticity index was *R. bulu* > *R. nyingchiense* > *R. wardii* > *R. nivale* > *R. aganniphum* (Figure 4c).

## 4 Discussion

### 4.1 Synergistic features among leaf anatomical traits of *Rhododendron*

Plant leaves often exhibit responses to environmental changes through phenotypic plasticity since they are directly exposed to the environment and they are sensitive to environmental change [25]. In the long term, environmental effects shape leaf anatomical traits as plants and their habitats form an integrated system [26]. This study assessed five *Rhododendron* species that displayed typical bifacial leaves with greater adaxial versus abaxial epidermal thickness, consistent with *Rhododendron ferrugineum* L. and *Rhododendron irroratum* Franch [27]. The adaxial epidermis is thicker than the abaxial epidermis and this structure facilitates gas exchange, enhancing photosynthesis capacity and stationary carbon capacity [28]. Significant interspecific variation was observed for leaf thickness, cuticle thickness, adaxial epidermal thickness, palisade parenchyma thickness, spongy parenchyma thickness, and ratio of palisade parenchyma to leaf thickness (Figure 2). Given the divergence in anatomical traits among the five species, adaxial epidermis, palisade, and spongy parenchyma may serve as anatomical markers for future phylogenetic analyses within *Rhododendron*. In general, leaf epidermis and mesophyll traits as well as leaf midrib and vascular traits perform different functions; however, they also work collaboratively and indispensably [12]. The correlation results suggest that there is some autocorrelation among the anatomical structures of the leaves of *Rhododendron* and demonstrate some synergistic changes. The synergistic nature of leaf anatomical structure reflects the fact that different leaf anatomical structures harmonize and constrain each other, increasing the plant’s ability to adapt to a variety of environments.

Papillae are products formed by the outward projection of the cell wall or cuticle of the plant epidermis [29]. The primary functions of papillae in plant leaves are antifungal penetration and water resistance [30]. Additionally, distinct papillae structures on *R. wardii* and *R. bulu* whose abaxial epidermal cells (Figure 3a1 and e1) align with leaf micromorphological observations of *Rhododendron myrtifolium* Ching ex Fang ex M.Y. He [31], the environment of the sampling location and the leaf maturity at the time of sampling could be factors in this outcome.

### 4.2 High plasticity is an ecological strategy for their adaptation to the Tibetan plateau environment

The plasticity index is a crucial measure of a plant’s capacity to adjust to its environment, and there is a positive correlation between a plant’s capacity for environmental adaptation and metrics [32–34]. As nutrition organs of plants, leaves are distinguished by extreme variability and plasticity in tissue structure. Each anatomical trait of *Rhododendron* leaves may have a specific adaptive strategy [35].

Greater combined plasticity was revealed in the spongy parenchyma thickness and epidermal parenchyma thickness (including the adaxial cuticle and abaxial epidermal), indicating that spongy parenchyma and epidermal structures are more adaptable to changes in their environments, it is the primary anatomical traits of *Rhododendron* that adapts to environmental change, but more data are needed to verify this results in the future. For example, meteorological data could be added in the future to verify this conclusion. Variations in the epidermal tissue’s plasticity index facilitate the absorption of light radiation by epidermal cells. Spongy parenchyma widths permit adequate carbon dioxide supply for photosynthesis [36]. The primary structure in plants for absorbing and storing water and nutrients is the leaf’s main vein [37]. Well-developed midrib tissue with larger cross-sectional areas can also ensure efficient transportation and an adequate supply of water and minerals and greater mechanical resistance against environmental stress [38]. Nonetheless, *Rhododendron* had a low plasticity index of the degree of midrib protuberant, these results are typically attributed to their differences in plant genetic characteristics and adaptability to the environment [39].

Variations between species in the integrated plasticity index of leaf anatomical traits indicate how sensitive they are to environmental changes [40]. According to related research, species with higher plasticity index tend to be more widely spread in a variety of settings because they can withstand more adversity [19,41]. *R. bulu* had the highest plasticity index. According to the field investigation, *R. bulu* grows widely between 3,000 and 5,300 m above sea level on the western slope of Shergyla Mountain. *R. aganniphum*, on the other hand, has the lowest integrated plasticity index, and it is found in the Shergyla Mountain summit zone over 4,200 m. This phenomenon is in line with the study findings, indicating that, among the five test plants, *R. bulu* is the most adaptable to heterogeneous habitats, however, the results need to be further analyzed in relation to environmental factors (meteorological data, soil data, etc.).

## 5 Limitation

A limitation of this study is the inability to incorporate meteorological data, which restricts a comprehensive understanding of the complex relationships between the environment and other factors.

## 6 Conclusions

Our study revealed that there are variations in the anatomical structure of the leaves of the five species of *Rhododendron* in Shergyla Mountain. These anatomical traits displayed a degree of association with one another, indicating synergistic adaptation. The high plasticity index indicates that the *R. bulu* has a significant ability to adapt to heterogeneous surroundings and can better occupy varied environments (low oxygen content, strong UV, etc.), becoming a widespread species. The results of this investigation provide beneficial suggestions for the selection and development of *R. bulu*. The study’s findings suggest that the level of plasticity index is an ecological tactic used by plants to adapt to their environment, suggesting that more research is needed on the physiological mechanisms of *Rhododendron* in the future.
